# Ulnar nerve injury after a comminuted fracture of the humeral shaft from a high-velocity accident: a case report

**DOI:** 10.1186/1752-1947-6-192

**Published:** 2012-07-10

**Authors:** Ritesh Pathak, Piyush Kalakoti, Duppala Venkateswara Prasad, Duppala Peeyuusha, Ragav Sharma

**Affiliations:** 1 Department of Orthopaedics, Rural Medical College, Loni, Maharashtra, 413736, India; 2 Rural Medical College and Pravara Rural Hospital, Loni, Maharashtra, 413736, India; 3 Naperville North High School, Naperville, IL, 60540, US

## Abstract

**Introduction:**

Injury to the ulnar nerve following humerus shaft fracture is a very rare entity because the ulnar nerve is well protected from the bone by muscle and soft tissue, and thus remains unaffected in these fractures. We report what is, to the best of our knowledge, the first case of ulnar nerve injury due to a comminuted humeral shaft fracture. The injury manifested and was diagnosed the day after a high-velocity accident. The paucity of related literature and the necessity for early diagnosis and subsequent treatment of such injuries in high-velocity accidents urged us to document this case.

**Case presentation:**

A 30-year-old Indian man presented to our Emergency Department after a road traffic accident. Our patient complained of right arm pain and the inability to move his extremity. The following morning he developed clawing. Nerve conduction studies on the peripheral nerves of his arm in addition to an X-ray confirmed the diagnosis of a possible injury to the ulnar nerve. Our patient was taken to our Operating Room for surgery, during which a fragment of bone was found abutting the ulnar nerve after penetrating his triceps. This fragment of bone was replaced and the fracture was reduced by open reduction and internal fixation using a dynamic compression plate and screws. Postoperatively, our patient received physical therapy and was discharged two weeks after surgery with no neurological deficit.

**Conclusions:**

This case emphasizes the urgency and importance of careful neurological examination of all the peripheral nerves supplying the arm in patients with a fracture of the shaft of the humerus. In the setting of injury to the arm in high-velocity accidents, a differential diagnosis of ulnar nerve injury should always be considered.

## Introduction

A fracture of the shaft of the humerus constitutes 3% to 5% of all fractures [1,2]. Radial nerve injuries are the most frequent nerve lesion associated with fractures of the shaft [3], with a reported incidence of radial nerve damage in almost 11.2% of cases [4-6]. Injury to the ulnar nerve after humerus shaft fractures is a very rare entity as the ulnar nerve is well protected from the bone by muscle and soft tissue, and thus remains unaffected in these fractures [3]. To the best knowledge of the authors, there has only been one case documented in the English medical literature of transection of the ulnar nerve after a fracture of the shaft of the humerus, which was explored four months after the injury. Moreover, no cases of ulnar nerve injury have been described that manifested immediately after a high velocity trauma accident. We report what we believe to be the first case of ulnar nerve injury due to a comminuted humerus shaft fracture, which manifested and was diagnosed the day after a road traffic accident. The paucity of literature and the necessity for early diagnosis and subsequent treatment of such injuries in high-velocity accidents urged us to document this case.

## Case presentation

**Figure 1:**
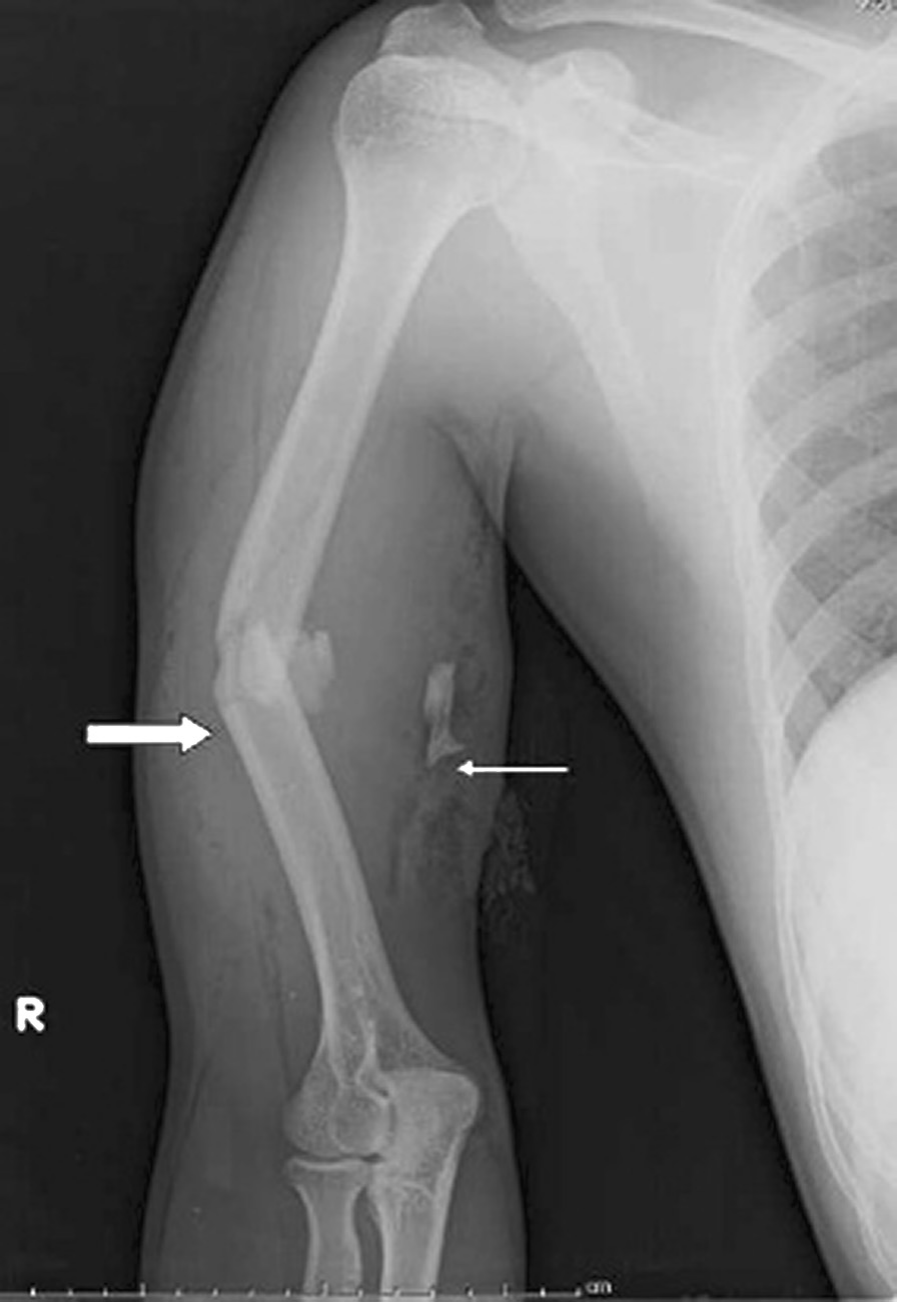
**Radiological finding.** Anteroposterior view of the right arm showing a displaced comminuted fracture of the mid shaft of the humerus (thick white arrow). A fragment of the bone can be seen displaced medially (thin white arrow).

A 30-year-old Indian man was brought to our Emergency Room after a road traffic accident, in which he was riding a motorcycle and was hit by a truck at a speed of approximately 50 miles per hour. Our patient complained of pain in his right arm and the inability to move it. An examination of his extremities revealed contusions over his right upper arm without any evidence of penetrating injury. There was marked swelling and tenderness in the middle part of his right arm associated with a closed injury. His cranial nerves were intact and no neurological deficits were present. No injures were found in his other limbs. In view of the above clinical findings, a radiograph of his right upper limb and a computed tomography (CT) scan of his head were suggested. While the CT scan did not reveal any abnormality, the radiograph of his right arm showed a unilateral displaced comminuted fracture of the mid shaft of his humerus (Figure 1). Our patient was then given first aid in the form of a U-slab and immediately transferred to our orthopedic ward for further evaluation. His laboratory results were normal. Liver and kidney function test values were within their normal range. An electrocardiogram did not show any abnormal features.

**Figure 2:**
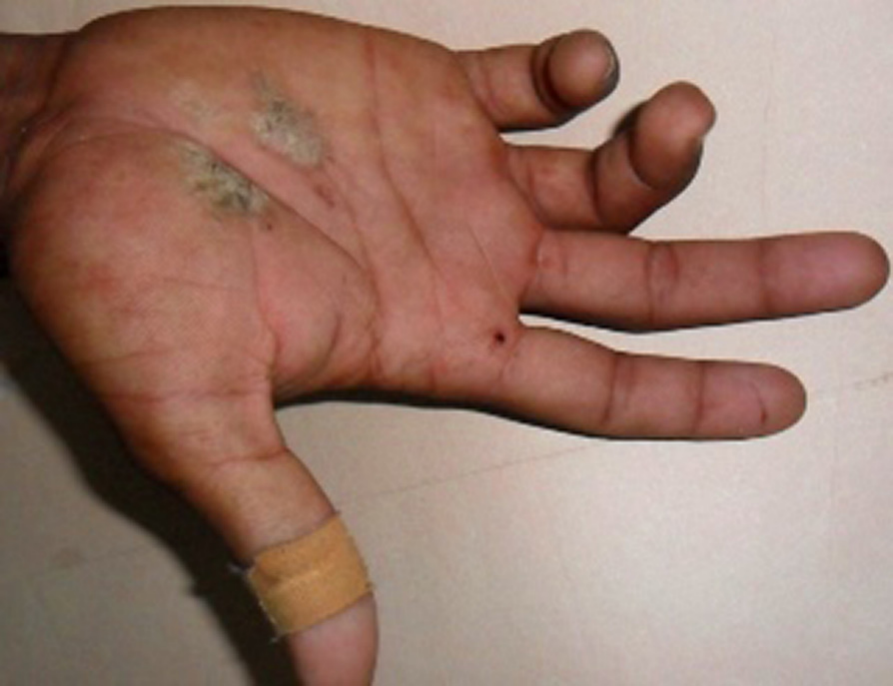
**Clinical photograph - clawing.** Hyperextension at the right metacarpophalangeal joints and flexion at the fourth and fifth proximal and distal interphalangeal joints with the inability to move the little and ring fingers.

Our patient complained of tingling and numbness in his right little and ring fingers the following morning. On examination, there was hyperextension at the metacarpophalangeal joints and flexion at the proximal and distal interphalangeal joints with an inability to move his little and ring finger (Figure 2). With the suspicion of an ulnar nerve injury due to the accident, a series of nerve conduction studies of his right upper arm nerves were suggested. The electrodiagnostic evaluation demonstrated a severe ulnar nerve conduction block along his arm with normal amplitudes of conduction along the median and the radial nerves.

**Figure 3:**
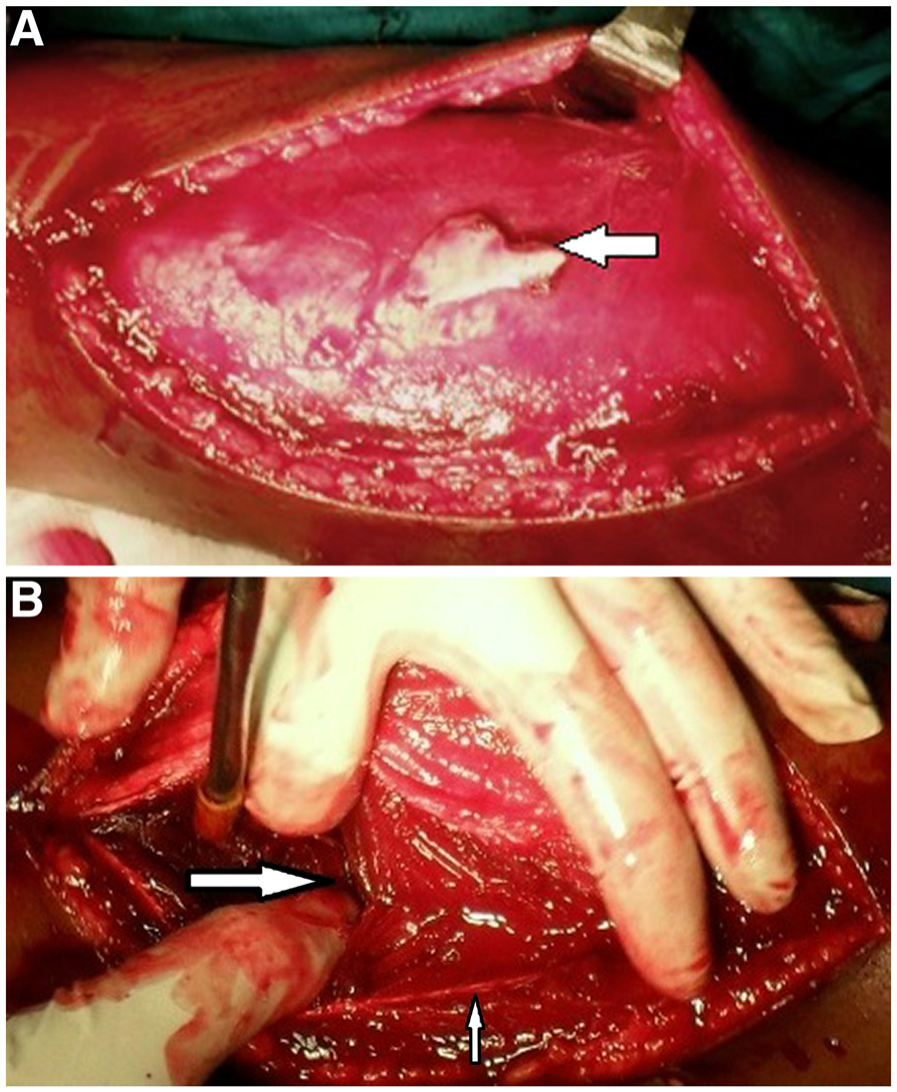
**Intraoperative photographs.** (**A**) Intraoperative photograph of the right arm showing the bone fragment (white arrow). (**B**) Finger showing a rent in the triceps muscle (horizontal arrow) caused by the comminuted fragment of the humerus. Ulnar nerve (vertical arrow) seen in relation to the rent.

Correlating the clinical findings with the above radiological and nerve conduction findings, a diagnosis of ulnar nerve injury after the fracture of the shaft of the humerus was made. Our patient was taken to the Operating Room for surgery, during which a fragment of bone was found abutting the ulnar nerve after penetrating the triceps muscle (Figure 3A,B). This fragment of bone was replaced and the fracture was reduced by open reduction and internal fixation using a dynamic compression plate and screws.

Postoperatively, the tingling and numbness of the little and ring finger gradually subsided and our patient received regular physiotherapy and was discharged two weeks after surgery. Our patient was advised to come regularly for follow-up every three months for a period of one year. Our patient first came for a follow-up appointment four months after discharge and told the attending surgeon about the recovery of the normal functioning of his right hand 14 weeks after surgery; confirming it to have been ulnar neuropraxia.

## Discussion

Humerus shaft fractures usually occur during high velocity trauma with the radial nerve being the most commonly injured [4-6]. In 1998, Stahl *et al*. reported a case of ulnar nerve palsy after a spiral fracture of the humerus shaft, which was supposedly the first case of its kind [3]. In our case, the ulnar nerve injury was due to the abutment of a fragment of the comminuted fractured humerus, in contrast to the case reported by Stahl [3]. In our patient, the symptoms of ulnar nerve injury manifested and were detected immediately the day after the accident. A fragment of the bone from the fracture of the humerus shaft was found to be the cause of the ulnar nerve injury. The nerve was pushed against the displaced bony fragment, which led to the neurological deficit on his right side. The abrupt stretching of the ulnar nerve by the forcible separation of the bone ends is implicated to be the cause of ulnar nerve injury in a fracture of the shaft of the humerus [7]. After the detection of the clinical manifestation and the subsequent confirmation on the nerve conduction studies, our patient was sent for surgery, which aimed to explore and reposition the bony fragment and fixate the fractured humerus so that the nerve was not further irritated by the bony fragment.

In 1990, Uchida and Sugioka [8] reported ulnar nerve palsy in six patients with a cubitus varus deformity seven years after supracondylar fractures of the humerus. Ulnar nerve palsy after percutaneous cross-pinning of supracondylar fractures [9] and after trampoline injuries [10] have also been reported but, to the best knowledge of the authors, this is the first case reported in the literature which documents a case of ulnar nerve injury due to comminuted fracture of the humerus shaft.

## Conclusions

This case emphasizes the urgency and importance of careful neurological examination of all the peripheral nerves supplying the arm in patients with a fracture of the shaft of the humerus. Similar cases occurring in other parts of the world should be documented as it would help promote the dictum of careful peripheral nerve examination and subsequent electrodiagnostic studies in such patients. In the setting of injury to the arm in high-velocity accidents, a differential diagnosis of ulnar nerve injury should always be considered.

## Consent

Written informed consent was obtained from the patient for publication of this case report and accompanying images. A copy of the written consent is available for review by the Editor-in-Chief of this journal.

## Competing interests

The authors declare that they have no competing interests*.*

## Authors’ contributions

RP, DVP and PK participated in the clinical diagnosis and patient care and follow-up. RP and DVP were the operating team of surgeons. PK contributed to sequence alignment and drafting of the manuscript. PK and RS made useful contributions to the review of the literature. PK, DP and RS participated in writing the discussion section. PK helped revise the manuscript. All authors read and approved the final manuscript.
